# Care-seeking and managing diabetes in rural Bangladesh: a mixed methods study

**DOI:** 10.1186/s12889-021-11395-3

**Published:** 2021-07-22

**Authors:** Hannah Maria Jennings, Joanna Morrison, Kohenour Akter, Hassan Haghparast-Bidgoli, Carina King, Naveed Ahmed, Abdul Kuddus, Sanjit Kumar Shaha, Tasmin Nahar, Kishwar Azad, Edward Fottrell

**Affiliations:** 1 Department of Health Sciences, University of York and Hull York Medical School, York, UK; 2 Institute for Global Health, University College London, London, UK; 3 Diabetic Association of Bangladesh, Dhaka, Bangladesh; 4 Department of Global Public Health, Karolinska Institutet, Solna, Sweden

**Keywords:** Type 2 diabetes, Care seeking, Bangladesh, Trust

## Abstract

**Background:**

Type 2 diabetes mellitus poses a major health challenge worldwide and in low-income countries such as Bangladesh, however little is known about the care-seeking of people with diabetes. We sought to understand the factors that affect care-seeking and diabetes management in rural Bangladesh in order to make recommendations as to how care could be better delivered.

**Methods:**

Survey data from a community-based random sample of 12,047 adults aged 30 years and above identified 292 individuals with a self-reported prior diagnosis of diabetes. Data on health seeking practices regarding testing, medical advice, medication and use of non-allopathic medicine were gathered from these 292 individuals. Qualitative semi-structured interviews and focus group discussions with people with diabetes and semi-structured interviews with health workers explored care-seeking behaviour, management of diabetes and perceptions on quality of care. We explore quality of care using the WHO model with the following domains: safe, effective, patient-centred, timely, equitable and efficient.

**Results:**

People with diabetes who are aware of their diabetic status do seek care but access, particularly to specialist diabetes services, is hindered by costs, time, crowded conditions and distance. Locally available services, while more accessible, lack infrastructure and expertise. Women are less likely to be diagnosed with diabetes and attend specialist services. Furthermore costs of care and dissatisfaction with health care providers affect medication adherence.

**Conclusion:**

People with diabetes often make a trade-off between seeking locally available accessible care and specialised care which is more difficult to access. It is vital that health services respond to the needs of patients by building the capacity of local health providers and consider practical ways of supporting diabetes care.

**Trial registration:**

ISRCTN41083256. Registered on 30/03/2016.

## Introduction

### Diabetes in Bangladesh

Type 2 diabetes (T2DM) is the third leading cause of mortality worldwide, with almost 80% of cases occurring in low and middle-income countries (LMICs) [1]. Additionally, the over 50 mortality associated with T2DM in LMICs is markedly higher than high income countries [2]. In many LMICs there are limited health budgets, high out-of-pocket expenditures and there is a lack of basic technologies and quality guidelines for professionals needed to assist people to manage their diabetes [2, 3]. Research into capacity and health care resources in LMICs are disproportionately low as compared to high income contexts [3]. Through this research we explore some of the issues as related to care-seeking in a low-income context. In Bangladesh diabetes affects an estimated 20 to 30% of the adult population either as intermediate hyperglycaemia or fully expressed T2DM [4, 5]. Despite the high prevalence, awareness about prevention, control and management are low [5, 6] and Bangladesh’s health system is ill-equipped to meet the increasing burden [7].

### Health systems in Bangladesh

Bangladesh has a pluralistic health system, with government, private and non-governmental organisations (NGOs) providing services. Government health care is provided at district level hospitals, upazilla (sub-district) level health complexes, union health and family welfare centres and ward level community clinics. Government services are often overstretched and the quality of care is variable with ineffective management, lack of resources, staff and equipment affecting service provision [8]. Meanwhile the private sector is poorly regulated and the non-formal sector largely consists of untrained, traditional and homeopathic practitioners [8]. Specialist diabetes care is provided by the Bangladesh Diabetic Association (BADAS) with centres in every district [9]. BADAS is a non-profit organisation that offers subsidised means-tested treatment and care for diabetes (with the poorest patients not paying for care). Patients can self-refer or are referred from government and private practitioners. There are physicians trained in diabetes screening and management, and the necessary testing and monitoring equipment is available [9]. However, access to these services may be limited by barriers such as distance, transportation and costs. While there is a national operational policy and strategy for diabetes in Bangladesh, they have only been partially implemented and basic technologies, medicine and training are often lacking in primary health care (PHC) [10]. The World Health Organisation (WHO) has developed a Package for Essential non-communicable (PEN) interventions specifically for PHC in low resource settings which incorporates strengthening health systems through training, equipment, medicines, referral and raising awareness [11]. PEN interventions have been piloted in Bangladesh, and while the Multisectoral Action Plan for Prevention and Control of NCDs references plans to scale-up PEN interventions [12] it is yet to be implemented.

### Evidence on quality and access to diabetes care in Bangladesh

There is limited research on access and quality of diabetes services in Bangladesh. Only one relevant qualitative study was identified [9]. Survey data on diabetes tends to concentrate on knowledge and be based in tertiary care facilities [13, 14]. The Bangladesh Demographic and Health Survey (BDHS), while dated (conducted in 2011), did gather information on fasting blood glucose and treatment of diabetes at a population level [6]. Analysis of this data found only 15% of female and 10% of males with diabetes have their blood glucose levels under control, with those in lower socio-economic groups significantly less likely to have their diabetes both diagnosed and treated [6]. Our research in rural Bangladesh found 75% of people with diabetes were unaware of their diabetic status, and 78% of those who were aware of their status did not monitor their blood glucose levels on at least a monthly basis [5]. A rare qualitative study exploring diabetic patient perspectives on care, found treatment and advice lacking in primary and non-specialist health facilities but when initial diagnosis and care was provided by specialists they tended to have a better understanding of diabetes management [9]. However, other studies at urban tertiary specialist centres [13, 14] found that knowledge and management of diabetes was also poor among their patients. Limited availability of services and costs of care were identified as barriers to access care [9]. While quality care should be both accessible and effective [15] the limited evidence from Bangladesh suggests that diabetic services can be difficult to access, treatment may not be effective and management of diabetes sub-optimal. With the exception of our published research [5] there is a general lack of up to date population-based data on diabetes, and details on care seeking patterns (type of treatment, glucose testing, non-allopathic treatment) are not available. Additionally there is a lack of qualitative data on access to diabetes care, care-seeking behaviour, experiences of care and the impact medicine adherence from a patient perspective. Furthermore none of the studies cited explore in detail the quality of care in diabetes services.

In order to address gaps in evidence this study examines care-seeking and management of diabetes in rural Bangladesh, it builds on and explores further some of our already published research looking at diabetes knowledge and more detailed care practices [5]. Additionally we explore quality of care using the WHO model with the following domains: safe, effective, patient-centred, timely, equitable and efficient [16].

## Methods

### Setting

This study draws on data collected as part of the D-Magic cluster randomised control trial (number ISRCTN41083256) testing two interventions (mHealth and a community based group intervention) on the prevention and management of diabetes in four rural upazillas of the Faridpur district in central Bangladesh [13]. Faridpur is approximately 200 km^2^ with a population of 1.7 million. It is mostly Bengali and 90% Muslim with farming being the main livelihood. At the time of data collection prevalence of T2DM was 10.5% and intermediate hyperglycaemia was 20.5% [5]. As in other parts of Bangladesh people seek care from a range of providers [8]. For diabetes this includes the ‘Diabetes Hospital’ (run by BADAS) at the district headquarters in Faridpur city. More locally health providers include government health complexes and community clinics as well as private ‘village’ doctors and traditional healers. Table 1 describes formal service providers available at upazilla level for diabetes care.

**Table 1:** Health facilities available at upazilla level for diabetes care^a^

Facility	Health workers	Training on diabetes	Equipment	Patients seen/month (number of people with diabetes seen) and opening hours
Bolmari Upazilla				
1 Upazilla health complex: Government	Doctors, nurses, medical assistants	Doctors received training on diabetes as part of their medical training	Blood monitoring and testing equipment available	5000–8000 (300) 24 h/day 7/7 days
4 community clinics: Government	Community Health Care Provider	No training	One of the community clinics reported having a glucometer	800–1000 (8–12 in two clinics) 9 am-3 pm 6/7 days in 1 clinic Not reported in 3 clinics
25 Pharmacies: Private	‘Village’ doctors (untrained health worker)	No training	11 of the pharmacies reported having glucometers	150–200 (2–10: in 10 pharmacies) 9 am-8 pm 7/7 days
Saltha Upazilla				
No upazilla level health complex				
1 Health and Family Welfare Centre: Government	Sub-assistant community medical officer, Family and welfare provider (2 staff total)	No training	No equipment for testing or monitoring diabetes	750–800 (No people with diabetes reported) 8–2.30 pm 6/7 days
3 community clinics: Government	Community Health Care Provider (typically 1/clinic)	No training	One of the community clinics had a glucometer	700–900 (No people with diabetes reported) 9-3 pm 6/7 days in 1 clinic Not recorded in 2 clinics
16 Pharmacies: Private	‘Village’ doctor (untrained health worker), 1 pharmacy also had a homeopathic practitioner Typically 1/ facility	No training	8 of the pharmacies reported having glucometers	50–200 (8–30 in pharmacies with glucometers) 9 am-8 pm 7/7 days
Nagarkanda Upazilla				
1 Upazilla health complex: Government	Doctors, nurses, medical assistants	Doctors received training on diabetes as part of their medical training	Blood monitoring and testing equipment available	10,000–15,000 (300) 24 h 7/7 days
1 Health and Family Welfare Centre: Government	Sub-assistant community medical officer (1 staff)	No training	No equipment for testing or monitoring diabetes	80–100 (No people with diabetes reported) 8–2.30 pm 6/7 days
3 Community clinics (cc): Government	Community Health Care Provider (Typically 1/clinic)	No training	No equipment for testing or monitoring diabetes	800–1000 (0) 9-3 pm 6/7 day in 1 clinic. 2 clinics – not reported
12 Pharmacies: Private	‘Village’ doctors (untrained health worker) (Typically 1/facility)	No training	7 of the pharmacies reported having glucometers	100–200 (15–30 in pharmacies with glucometers) 9 am-8 pm 7/7 days
Modhukali Upazilla				
1 Upazilla health complex: Government	Doctors, nurses, medical assistants	Doctors received training on diabetes as part of their medical training	Blood monitoring and testing equipment available	10,00–15,000 (300) 24 h 7/7 days
1 Health and Family Welfare Centre: Government	Sub-assistant community medical officer, Family and welfare provider (2 staff in total)	No training	No equipment for testing or monitoring diabetes	800–1000 (No people with diabetes reported) 8–2.30 pm 6/7 days
3 Community clinics: Government	Community Health Care Provider (typically 1/clinic)	No training	One of the community clinics had a glucometer	800–1000 each (8–10 in one clinic, not reported in others) 9-3 pm 6/7 days in 1 clinic Not reported in two clinics
16 Pharmacies: Private	‘Village’ doctors (untrained health worker) (typically 1/facility)	No training	8 of the pharmacies reported having glucometers	50–200 (5–25 in pharmacies with glucometers) 9 am-8 pm 7/7 days

^a^ Data collected by BADAS field staff in 2016. Data was not collected from the non-formal sector (such as village doctors practising from home and alternative practitioners)

### Study aim, objectives and design

We aim to understand the multiple factors affecting care-seeking and diabetes management in rural Bangladesh. This will be achieved through three objectives: 1. Examine care seeking practices and management of diabetes focusing on gender and wealth quantitative data; 2. Examine patient experiences of access and quality of care for diabetes; 3. Qualitatively explore care-seeking practices and medication adherence.

**Fig. 1:**
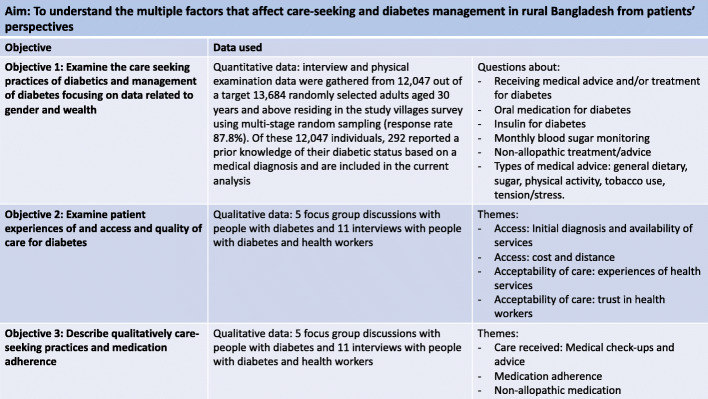
study objectives and corresponding data used

We used quantitative data from the trial baseline survey, and qualitative data collected as part of the formative phase of the trial, both collected in 2016. We used a partially mixed concurrent equal study design, whereby data collection was concurrent and carried equal weight and integration of the data was only at the interpretation phase [17]. The qualitative data complements the quantitative data on care-seeking by exploring in greater depth the reasons behind patterns of care-seeking and variations in medication adherence. Figure 1 shows the data used for each research objective.

### Quantitative data collection, management and analysis

The survey was conducted among a target random sample of 13,684 adults aged ≥30 years in 96 villages in the four upazillas, covering a population of approximately 125,000. Sample size was determined by trial requirements described in detail elsewhere [18] and participants were randomly selected using two-stage random sampling from a register of all household and eligible participants. Anthropometric data, fasting blood glucose and 2 h post-load glucose levels were taken from all survey participants. Additionally sociodemographic and behavioural data of all consenting individuals were collected through interviews using a structured survey instrument adapted from the WHO STEPwise tool [19] and the 2011 Bangladesh Demographic and Health Survey [4]. The tool was piloted, adapted and data were collected by 16 pairs of researchers who were recruited locally and received 1 month’s training on survey methods. The interviews took approximately 1 h and participants who knew they were diabetic were asked about complications, care-seeking, medication and testing. For our analysis we used data from individuals identified with diabetes and explored care-seeking among 292 people living with diabetes who reported that they had been previously diagnosed as diabetic by a medical professional.

We conducted a descriptive multi-variate analysis using Stata v.13, summarising practices of people with diabetes regarding medical advice, medication, complications and the use of non-allopathic medicine. Associations between diabetic practices and gender and socioeconomic wealth quintiles were assessed using multivariate logistic regression. Households were categorised into five socioeconomic quintiles using a wealth index created from principle components analysis of household ownership of assets, land ownership, sanitation and housing characteristics [20]. Demographic features (sex, wealth, age, occupation, education, marital status, religion) were adjusted for in the multivariate models, as we reason that all these features may influence our results.

### Qualitative research data collection, management and analysis

Data were collected in three out of four upazillas. Data saturation was reached after collecting data in three upazillas, and the upazillas were similar in terms of rates of diabetes, services available and population. Data saturation was assessed through reviewing emerging themes following the data collection. This paper reports on the data from 6 interviews (n3 women, n3 men) and 5 FGDs (n3 women, n2 men) of people with diabetes and 5 interviews with local health workers (n2 private ‘village doctors’, n1 NGO and n2 government) who provided care to people with diabetes. By triangulating data (i.e. using different data collection methods) we increased the reliability of the data [21]. Participants were purposively sampled and recruited from villages in each of the upazillas. An experienced qualitative researcher approached participants and invited them to participate in the study. She was not familiar with the village and therefore it was challenging for her to find participants meeting criteria. After finding a few participants with diabetes, she used snowball sampling to locate other participants with diabetes, who were then invited to participate. Respondents were aged between 30 and 60 and there was a balance of better-off and lower socio-economic groups as assessed by observing house construction materials and occupation.

**Fig. 2:**
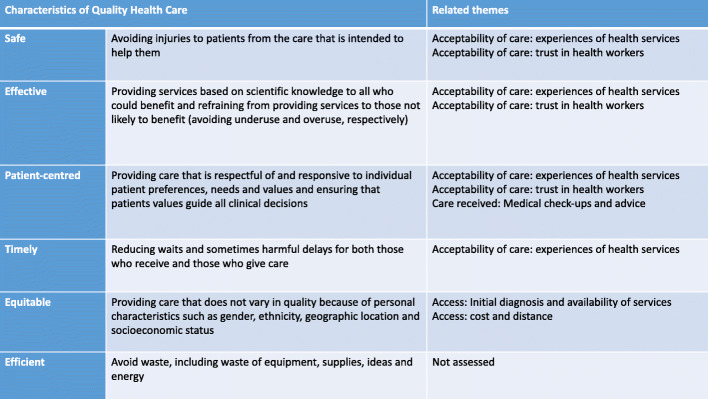
WHO characteristics of quality care and related themes

The interviews and FGDs were conducted in Bangla using topic guides developed on the basis of the research objectives, literature reviews and COM-B behaviour change theory [22]. Topic guides were developed in English, translated into Bangla and piloted in Dhaka. The FGDs and interviews were recorded and transcribed from Bangla into English. The translations were checked through listening to the recordings and back-translation. A coding framework was agreed on by three qualitative researchers (HJ, JM and KAk) and organised in NVIVO 12. The data related to care-seeking was analysed through a framework matrix [23] by HJ. The agreed themes related to care seeking were charted and summarised according to each interview and FGDs, this enabled a description of the themes and comparisons within and between the transcripts which is reported on. Themes were analysed and organised according to the research objectives (Fig. 1). For objective 3 quality was examined using the WHO model, themes relating to the characteristics in this model were mapped and analysed accordingly (Fig. 2: WHO characteristics of quality of care and related themes).

The data collection and analysis was completed by three experienced qualitative researchers (KAk, HJ, JM). The researcher who conducted the interviews and FGDs is a woman, Bengali, middle class, bilingual (in English and Bangla) and based in Dhaka; she has worked in health research for many years and has spent time in rural Bangladesh. The other researchers involved in the analysis have lived many years in South Asia, one in Bangladesh and is bilingual (English and Bangla). The three researchers discussed and reflected on their own status and perceptions of the findings.

### Ethics

All research participants gave written or thumb-print informed consent. The research received ethical approval from ethics committees at University College London and BADAS.

## Results

### Quantitative results: care seeking practices of people with diabetes in rural Bangladesh by gender and socioeconomic status

Survey data were collected from 12,140 individuals out of a targeted 13,684. Non-responders were more likely to be men than women (15.7% vs 7.0%) and were younger (mean 46.5 years vs 47.7 years) [5]. Ninety-three individuals had data missing on diabetic status and 2 had missing data on occupation, hence were excluded from the analysis. The proportion of people with diabetes identified through our blood glucose testing who reported a prior medical diagnosis of diabetes from a medical professional was 25.0% (*N* = 292/1225). Table 2 illustrates the sociodemographic distribution of people with a prior diagnosis of diabetes.

Women living with diabetes had 47% lower odds of having had a prior medical diagnosis compared to men (adjusted odds ratio (95% confidence interval) 0.53(0.33–0.86)). Education was strongly associated with one’s awareness of their diabetic status, with those who had completed at least primary education more than twice as likely to have had a prior medical diagnosis than those with no formal education (AOR(95%) 2.30(1.74–3.12)). Crude associations observed with wealth and occupation appeared to be confounded by other sociodemographic factors, including gender, education and age (Table 2).

**Table 2:** Sociodemographic distribution of people with a prior medical diagnosis of diabetes among people living with diabetes in Faridpur

	No prior diagnosis of diabetes (N (%))	Prior diagnosis of diabetes (N (%))	Crude odds ratio (95%CI)	Adjusted^a^ odds ratio (95% CI)
Sex				
Male	352 (70.7%)	146 (29.3%)	Ref	Ref
Female	581 (79.9%)	146 (20.1%)	0.61 (0.46–0.79)	0.53 (0.33–0.86)
Wealth				
Most poor	193 (78.8%)	52 (21.2%)	Ref	Ref
Very poor	208 (84.9%)	37 (15.1%)	0.66 (0.41–1.05)	0.60 (0.37–0.96)
Poor	203 (82.9%)	42 (17.1%)	0.77 (0.49–1.05)	0.59 (0.37–0.94)
Less poor	181 (73.3%)	66 (26.7%)	1.35 (0.89–1.05)	0.89 (0.57–1.39)
Least poor	148 (60.9%)	95 (39.1%)	2.38 (1.60–3.55)	1.22 (0.77–1.92)
Age group				
30–39	256 (86.8%)	39 (13.2%)	Ref	Ref
40–49	230 (76.9%)	69 (23.1%)	1.97 (1.28–3.03)	2.20 (1.40–3.44)
50–59	181 (69.1%)	81 (30.9%)	2.94 (1.92–4.50)	3.10 (1.94–4.90)
60–69	173 (70.6%)	72 (29.4%)	2.73 (1.77–4.22)	2.76 (1.68–4.54)
70 and up	93 (75.0%)	31 (25.0%)	2.19 (1.29–3.71)	2.09 (1.11–3.94)
Occupation				
Unemployed/ retired/housewife	612 (76.8%)	185 (23.2%)	Ref	Ref
Manual	213 (82.6%)	45 (17.4%)	0.70 (0.49–1.00)	0.41 (0.24–0.70)
Professional/Business	108 (63.5%)	62 (36.5%)	1.90 (1.33–2.70)	0.75 (0.44–1.28)
Education				
No formal education	483 (82.0%)	106 (18.0%)	Ref	Ref
Incomplete primary	176 (79.3%)	46 (20.7%)	1.20 (0.81–1.75)	1.32 (0.87–2.00)
Completed at least primary	274 (66.2%)	140 (33.8%)	2.33 (1.74–3.12)	2.13 (1.47–3.80)
Marital status				
Unmarried	137 (77.8%)	39 (22.2%)	Ref	Ref
Married	796 (75.9%)	253 (24.1%)	1.12 (7.61–1.64)	0.98 (0.62–1.56)
Religion				
Non-Muslim	88 (65.7%)	46 (34.3%)	Ref	Ref
Muslim	845 (77.5%)	246 (22.6%)	0.56 (0.38–0.82)	0.71 (0.47–1.08)

^a^adjusted for all covariates

Table 3 shows the association of gender and wealth with care seeking, medication practices and complications among people with a prior diagnosis of diabetes. 86.3% of people with a prior diagnosis of diabetes reported receiving medical advice and/or treatment within the past 30 days. 80.5% of people with a prior diagnosis of diabetes reported taking oral medication for diabetes and over 50% reported taking insulin. Only 21.9% of people with a prior diagnosis of diabetes reported having their blood glucose tested in the last month and 73.6% reported experiencing complications. Reporting to have ever used non-allopathic medicine was 9.2%. 17.1% of women compared to 26.0% of men reported having their blood sugar tested, and women were less likely to take insulin for diabetes than men (AOR 0.53(95%) (0.23–1.22).

Receipt of treatment, advice and monthly blood glucose testing was more frequently reported by those in the least poor group than the most poor and very poor groups. These associations were not statistically significant, although given the fairly large differences between groups and apparent dose response trend with wealth, the possibility of type II errors due to small sample size must be noted. A significant association between wealth and insulin use and blood sugar testing was observed with the most wealthy group being considerably more likely to receive insulin (AOR(95%CI) 3.32(1.34–8.05)) and have their blood sugar tested in the last month (AOR 1.90(95%) 1.90(0.72–4.94)) than the least poor groups (Table 3).

**Table 3:** Care-seeking, medication and diabetic-related complications among known people with a prior diagnosis of diabetes according to gender and wealth^a^

		Receives medical advice and/or medication			Takes oral medication for diabetes			Takes Insulin for diabetes			Blood sugar tested (in the last month)			Ever used non-allopathic treatment			Experiences complications		
		N (%)	Crude OR (95% CI)	Adjusted OR^b^ (95% CI)	N (%)	Crude OR (95% CI)	Adjusted OR^b^ (95% CI)	N (%)	Crude OR (95% CI)	Adjusted OR^b^ (95% CI)	N (%)	Crude OR (95% CI)	Adjusted OR^b^ (95% CI)	N (%)	Crude OR (95% CI)	Adjusted OR^b^ (95% CI)	N (%)	Crude OR (95% CI)	Adjusted OR^b^ (95% CI)
Sex	Men [*n* = 146]	128 (87.7%)	Ref	Ref	119 (81.5%)	Ref	Ref	78 (53.4%)	Ref	Ref	38 (26.0%)	Ref	Ref	17 (11.6%)	Ref	Ref	107 (73.3%)	Ref	Ref
	Women [*n* = 146]	124 (84.9%)	0.79 (0.41–1.55)	0.87 (0.26–2.88)	116 (79.5%)	0.88 (0.49–1.57)	1.03 (0.38–2.79)	72 (49.3%)	0.85 (0.54–1.34)	0.53 (0.23–1.22)	26 (17.1%)	0.62 (0.35–1.08)	0.95 (0.36–2.50)	10 (6.8%)	0.56 (0.25–1.30)	0.80 (0.17–3.76)	108 (74.0%)	1.04 (0.62–1.74)	1.10 (0.45–2.71)
Wealth	Most poor [*n* = 52]	43 (82.7%)	Ref	Ref	43 (82.7%)	Ref	Ref	22 (51.2%)	Ref	Ref	9 (17.3%)	Ref	Ref	5 (11.6%)	Ref	Ref	40 (76.9%)	Ref	Ref
	Very poor [*n* = 37]	30 (81.1%)	0.90 (0.30–2.67)	0.95 (0.31–2.94)	27 (73.0%)	0.57 (0.20–1.57)	0.58 (0.20–1.68)	17 (46.0%)	1.15 (0.50–2.71)	1.42 (0.58–3.44)	4 (10.8%)	0.58 (0.16–2.05)	0.54 (0.15–2.00)	4 (13.3%)	1.14 (0.28–4.57)	0.94 (0.22–4.03)	30 (81.1%)	1.29 (0.45–3.66)	1.41 (0.48–4.15)
	Poor [*n* = 42]	34 (81.0%)	0.89 (0.31–2.55)	0.99 (0.32–3.06)	31 (73.8%)	0.59 (0.22–1.59)	0.64 (0.22–1.90)	18 (42.9%)	1.02 (0.45–2.32)	1.38 (0.57–3.33)	5 (11.9%)	0.65 (0.20–2.10)	0.56 (0.16–1.93)	5 (14.7%)	1.27 (0.34–4.72)	0.81 (0.20–3.31)	27 (64.3%)	0.54 (0.22–1.33)	0.68 (0.26–1.78)
	Less poor [*n* = 66]	59 (89.4%)	1.76 (0.61–5.11)	1.77 (0.55–5.67)	54 (81.8%)	0.94 (0.36–2.44)	0.94 (0.33–2.66)	37 (56.1%)	1.73 (0.83–3.63)	2.68 (1.17–6.15)	16 (24.2%)	1.53 (0.61–3.81)	1.30 (0.47–3.50)	7 (11.9%)	1.12 (0.33–3.74)	0.81 (0.21–3.13)	46 (69.7%)	0.69 (0.30–1.59)	0.75 (0.30–1.87)
	Least poor [*n* = 95]	86 (90.5%)	2.00 (0.74–5.40)	1.72 (0.55–5.39)	80 (84.2%)	1.11 (0.45–2.76)	0.96 (0.34–2.70)	56 (59.0%)	1.96 (0.99–3.89)	3.23 (1.43–7.27)	30 (31.6%)	2.21 (0.95–5.10)	1.90 (0.72–4.94)	6 (5.2%)	0.63 (0.18–2.19)	0.41 (0.10–1.73)	72 (75.8%)	0.94 (0.42–2.09)	1.06 (0.42–2.64)
Total (%)	292 (100%)	252 (86.3%)			235 (80.5%)			150 (51.3%)			64 (21.9%)			27 (9.2%)			215 (73.6%)		

^a^ The table does not report on the percentage (and *N* values) who responded “no” or missing data. There was, however, no missing data

^b^adjusted for sociodemographic covariates (sex, wealth, age group, occupation, education, marital status, religion)

**Fig. 3:**
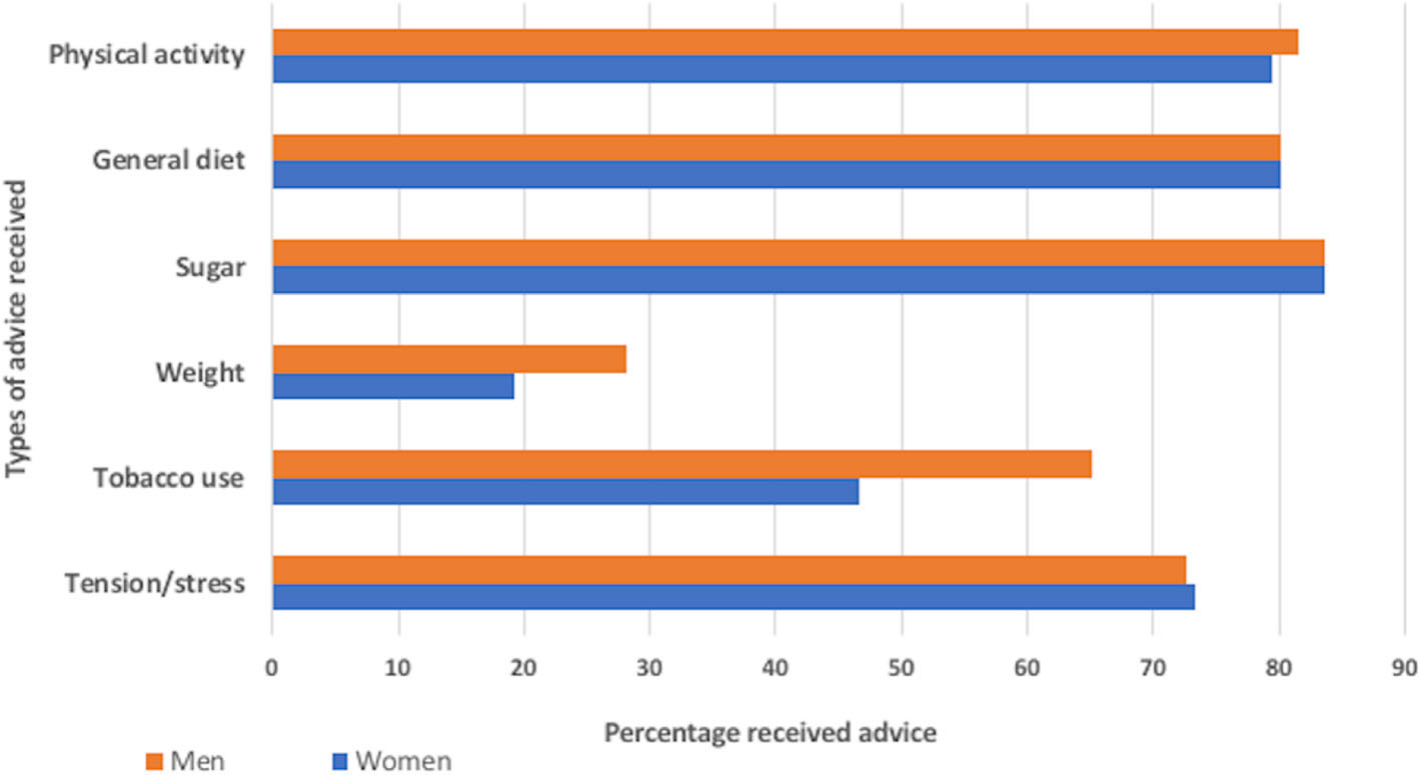
Types of advice received by people with a prior diagnosis of diabetes

In addition to treatment most people with a prior diagnosis of diabetes reported receiving behavioural advice from a medical professional regarding the management of diabetes, summarised in Fig. 3: types of advice received by people with diabetes. Advice about diet and exercise were the most common types of advice, received by over 80% of people with a prior diagnosis of diabetes, and 72.9% of people received advice about stress. Specific advice relating to weight control and tobacco were more frequently reported by more men than women, but differences were not statistically significant (results not shown).

### Qualitative results: experiences of care seeking (access and quality)

#### Access: initial diagnosis and availability of services

Receiving a diabetes diagnosis was a lengthy process for most. While some participants described going to a health professional due to feeling very unwell and/or experiencing the symptoms of diabetes which led to testing and a diagnosis, others reported being diagnosed when in hospital for another health condition or because of complications related to diabetes. They described being very sick and a long wait before being tested for diabetes.

*“My doctor prescribed medication* [for fever and weakness] *but my health didn’t improve..…..so I went to Faridpur, but the doctor was not there. So I went to another doctor who did blood tests. He later told me I had typhoid, and I was also suffering from diabetes”*

Men’s FGD(013)

Most participants received a diagnosis in Faridpur (the district headquarters), though they frequently relied on non-specialist local health professionals for on-going advice and check-ups. Almost all had reported having visited more than one facility. Table 1 describes the available health services at upazilla level and shows a lack of training in diabetes care and blood monitoring equipment. The community clinics offer free care but lack glucometers and though officially open for 6 h a day participants reported they were frequently closed. Pharmacies were most easily available due to their longer opening hours (12 h/day, 7 days/week). However, they are not free, health workers there often lack training and participants reported that glucose testing strips and insulin were not always available. Local private village ‘doctors’ would also often be consulted either at pharmacies or in their private offices.

Health workers confirmed there were few diabetic specialists. Four of the five interviewed were able to test for diabetes through random blood sugar tests, and reported that they would refer patients who had ‘high’ blood glucose readings (generally above 10) to specialists. Three said they could prescribe medication for diabetes, and all five were able to provide general behavioural advice. However, they all said they had very little training and lacked appropriate resources to care for diabetes patients.

#### Access: cost and distance

When seeking specialist care most participants found that travelling to and from the diabetic hospital in Faridpur, usually by bus, was time consuming, difficult and uncomfortable.

*“The visits are difficult for me. My home is at the far end of Nagarkondha and I have to go to Faridpur*. *The travel causes me much pain”*

Diabetic man SSI026

The journeys were not only inconvenient, but add to the costs of diabetes care; return costs ranged from 100 taka (2USD) to 2000 taka (40 USD) dependent on the distance and mode of transport. Costs of check-ups, tests and medicines were also a major concern. The Diabetic Hospital does subsidise treatment dependent against income and the government upazilla health complex provide free consultations and blood glucose tests, however they are not available in every upazilla and can still be far to travel.

*“It costs 50 taka* [approx. 1USD] *to go to Faridpur* [one way]. *It is difficult for a poor person to spend 100 taka* [2USD] *on a check-up. If a farmer or a day labourer goes to the hospital s/he does not earn their livelihood that day. They also pay for the doctor’s bills, tests and medicine”*

Health worker SSI010

There were important gender differences in regards to travel as several women reported needing an escort (usually by a man relative) when travelling outside the home. Not having an escort could mean that they would miss treatment and/or check-ups,

*“I was given a course of treatment* [at the diabetic hospital] *and felt a little better and came back home. I did not go for further check-ups after that as my husband was abroad.”*

Woman’s FGD(021)

#### Acceptability of care: experiences of health services

When discussing the quality of care participants spoke primarily about the diabetic hospital in Faridpur and health workers close to the community. The diabetic hospital was generally reported to have good quality care in terms of treatment and guidelines.

*“the diabetes hospital is the best option to receive treatment for diabetes. They will give proper guidelines and a diet plan”*

Woman diabetic SSI015

Views on health workers at the diabetic hospital varied somewhat. For example in an FGD(021) a participant reported that she felt the quality of the diabetes hospital was exceptional and was impressed by the care she received from the doctors, in another FGD(013) two participants felt not enough personalised care was shown with the focus being on medication and treatment. Despite some varied views there was a general consensus that the doctors at the diabetes hospital could diagnose and treat diabetes. However, most patients found that the hospital was crowded and waiting times were lengthy.

*“It takes a long time. They take blood, urine. So we have to wait…sometimes it takes a whole day.”*

Women’s FGD029

Upazilla level health complexes, if available, were closer than the diabetes hospital and had more expertise and equipment than local providers – however, they were also crowded with long waiting times to see a doctor. Local health workers such as village doctors, were reported to be more likely to spend time with patients in less crowded conditions. Some problems with health workers, in different facilities were described, such as giving the wrong medication (FGD21) and ‘overtreating’ (FGD13). People therefore shopped around and would go back to health workers they trusted.

#### Acceptability of care: trust in health workers

Experiences of care and where one went for care were heavily influenced by having a trusted health worker. A trusted health worker was described as being highly regarded by others, experienced and not motivated by money. Trusted health workers could build up a reputation and participants would be more likely to visit them.

*“I came to know of him* [her doctor] *as everyone in this area likes him. We did not know anyone for diabetes. But people suggested that we go to him”*

Woman diabetic SSI025

In contrast health workers that were seen to prioritise business over quality of care were not trusted. There was much scepticism about health services and health workers as highlighted in a men’s FGD,

*Participant 1: The most corruption right now is in the health department.*

*Participant 2: Yes…among the doctors….they are taking money that you do not even understand.*

*Participant 1: this is 500 taka and that is 2700 taka, they take money for nothing*

Men’s FGD(023)

Health workers also recognised the importance of their behaviour and trust in their relationship with the patient and echoed patient concerns of health workers who prioritise business over patients’ wellbeing.

*“if a patient gets well because of me then that is important to me as a doctor. And if you talk nicely with the patients they are able to understand. It is due to this that I have attained a place here”*

Health worker SSI028

Most trusted health workers were physically close to participants, although there were some exceptions with a few participants travelling some distance to visit a worker that they trusted (FGD011, FGD023, FGD021). Gender features in decisions as more women reported seeking and returning to a trusted health worker than men.

### Qualitative results: care-seeking practices (check-ups, advice and medication)

#### Care received: medical check-ups and behavioural advice

All participants reported going to medical facilities for diabetes care. Following an initial diagnosis of diabetes, participants were given behavioural advice and most took oral medication or insulin to manage their diabetes. They were expected to attend regular ‘check-ups’ where their blood sugar would be monitored, medication reviewed and advice given. While several participants reported visiting a health provider on a ‘regular’ basis (one-three monthly) regarding their diabetes, others did so rarely or only had a check-up when they felt unwell (FGD11, FGD29). Two participants monitored their blood sugar levels at home rather than go for a check-ups (FGD013, SSI015) and others reported they had gone regularly for check-ups following diagnosis but they no longer did (FGD013, SSI015).

Triangulating with quantitative findings, the most common advice participants reported receiving was basic advice regarding diet and physical activity; the general medical advice reported was to increase physical activity through walking, reduce sugar intake, have smaller portions and replace one rice meal a day with *ruti* (flatbread)*.* Participants would talk about the advice as ‘rules’ that should be followed,

*“We maintain rules, he* [the doctor] *tells me ‘eat a little, try to eat* ruti *two times a day and do some walking…and take medicine’”*

Woman’s FGD11

#### Medication adherence

All but two participants reported being prescribed medication for their diabetes. While a minority said they took medication as advised, most highlighted difficulties with medical adherence. Reasons for difficulties included structural constraints such as costs, side-effects from medication and a lack of trust in those prescribing.

A few participants reported skipping doses of medication due to forgetting or lacking time (SSI04, SSI016, SSI026). A lack of availability of insulin locally was highlighted as a problem in a woman’s FGD(021), meaning she was not able to take insulin regularly. Several participants said they felt unwell after taking medication or reported side effects, leading them to stop taking or reducing medicine (SSI03, FGD021). Several also reported they stopped or reduced medication when they felt well as they believed they no longer needed it. The cost of medication was also an important factor in whether medication was taken regularly,

*“But as I am a poor woman, sometimes if I feel good I don’t buy the medicines and I do not take them”*

Woman participant SSI025

While most of the participants did take at least some of the medication advised by doctors, many participants did not trust the judgement of the health professional regarding the exact doses of medication and would therefore adjust their medication accordingly (FGD013, FGD021).

#### Non-allopathic medication

The use of alternative remedies for diabetes such as herbal medicine and plants were reported to be common place (SSI015, SSI025, SSI016, all FGDs). A minority of participants had tried herbal medicines for diabetes and found them to be unhelpful or caused side effects (such as vomiting) leading to them no longer taking them. However, most participants who consumed medicinal plants or “bitter leaves” did believe they helped, at least in part.

*“taking bitter leaves may control diabetes. The juice from the leaves work, but not fully.”*

Men’s FGD13

While medicinal plants may be advised by traditional practitioners it was more common for them to be recommended by friends and family. “Bitter leaves” and other medicinal plants to help control diabetes were prepared and consumed in different ways – either specifically for medicinal purposes (such as the sap, raw or soaked in water) or more generally as part of a meal. One participant (SSI025) reported that she often consumed medicinal plants instead of medication, as she cannot always afford medication. However, more commonly plants were taken alongside allopathic medication to manage diabetes. Reasons given for their use were pragmatic as they were believed to help control diabetes, and they were seen as complementary to allopathic medicine. One of the health workers indicated his somewhat pragmatic and pluralistic approach to the type of medications taken to control diabetes that was reflected in our data,

*“If one is active and takes medicine – allopathic, homeopathic or herbal – then your physical condition will be good”*

Health care worker SSI010

## Discussion

The findings of our study highlight the challenges and implications of care-seeking for people with diabetes in rural Bangladesh. The quantitative data indicates that people with diabetes in Faridpur do seek care and receive advice and medication to manage their diabetes, however it does not capture the quality of care received. It also reveals differences across gender and wealth groups, and high levels of complications among people with a prior diagnosis of diabetes. The qualitative data highlights barriers to accessing care and the knock-on impact on medication adherence and ‘doctor shopping’ among people with diabetes. Gender, familial support, wealth and trust all impact where people seek care and how they manage diabetes. While this research is context specific to rural Bangladesh, it is relevant to global debates on care-seeking and care quality, medication adherence and doctor shopping.

### Diabetes care, access and quality

Obtaining a diabetes diagnosis and receiving consistent quality diabetes care is often problematic in Bangladesh. Only 25% of people with diabetes in our survey had received a prior diagnosis which could be due to delayed care seeking and/or professionals’ missing a diagnosis. The often lengthy process to diagnosis described in the qualitative results indicates a lack of awareness about diabetes from both patient and health provider perspectives. It also means that people with diabetes are usually at an advanced stage in their diabetes by the time they receive a diagnosis; this is also evident from the high proportion of complications of diabetes experienced and high levels of insulin use among people with a prior diagnosis of diabetes reported in the quantitative results (73.6 and 51.3% respectively). The quantitative results revealed that lower socioeconomic groups were less likely to receive treatment and advice and were significantly less likely to have their blood glucose tested and take insulin – previous analysis of the survey data reveal costs of care disproportionately affect the poorest strata with diabetic patients spending up to 20% of their monthly expenditure on diabetic care [24]. The qualitative results find costs affected where one went for care and medication adherence. Access to more specialised care was particularly limited due to barriers of distance, cost and time whereas local care provided by non-specialists was more accessible. Our findings are similar to other studies in low-income contexts. Research in urban India found the high costs of care was a major factor in diabetic patients changing medical facilities [25] and a study looking at diabetes management in urban Tanzania found that people of low socioeconomic status faced difficulties engaging with diabetes services consistently [26].

Experiences and perceptions of care varied according to where one seeks care. Drawing on the WHO characteristics of quality health care (*safety, effectiveness, patient-centred, timely,* and *equitable*) our findings reveal overall the quality is patchy at best in regards to each characteristic; though it varies locally, at district and upazilla level*.* While most participants did not express concerns about care being un*safe*, there were some reports of incorrect prescribing and over-testing. An unregulated private sector and overstretched government sector does mean services can go unchecked. At a district level participants generally concurred that they were able to receive *effective care* from the diabetic hospital as specialists and testing facilities were available there which follow international guidelines. However, at a local village level the lack of specialist knowledge, training and support for non-specialists as well as a deficit in and equipment for testing and monitoring diabetes meant that effective care is compromised locally. Participants expressed distrust towards to some health professionals locally and at a district level, raising concerns of profit being prioritised over patient welfare and unnecessary testing of patients. Both over and under-testing of patients has been reported in Bangladesh [27]. A lack of trust in healthcare workers also indicates that care is not always perceived as effective. Furthermore ongoing trust is key to *patient-centred* health care [28, 29]. At busy health facilities participants reported having little time with the doctor, thus it was unlikely care would be personalised. Indeed participants would return to doctor’s who were familiar with them and spent more time with them. However there was little acknowledgement of individual patient needs in the interviews, this is evident in the lack of discussion around medication adherence. Poor medication adherence often indicates a lack of integrated and patient-centred care [30]. Crowded conditions, short consultation times and long waiting times meant care was not described as *timely.* While reduced in more local health facilities, they often lacked specialist knowledge and blood monitoring equipment and diagnostic tests. The study indicates that diabetes care was not *equitable,* despite some level of care being available to all, access clearly varies according to geographical location and socioeconomic status. Furthermore, gender considerations from the health-supply side were not evident. Women had greater challenges in travelling to receive care and were more likely to seek care locally. A lack of gender considerations in both public and private health provisions is reported elsewhere in South Asia [31].

### Managing diabetes and shopping for care

As a result of barriers to access and the variable quality participants ‘shop around’ for care. Where one seeks care may vary according to circumstances, the money they have available and family support. Distance, costs and crowded conditions deterred people from going to specialists and trust was a crucial factor in where people (particularly women) sought care. Evidence from other settings has highlighted the importance of trust in decision making about care [32]. Indeed the phenomenon of ‘doctor shopping’ for the treatment of chronic conditions is a common internationally [33]. The negative consequences of ‘doctor shopping’ include a loss of a continuity of care, risks of multiple drugs being inappropriately prescribed [33], adding to excess costs and loss in efficiency of care [25]. While a study in urban India exploring pluralism found diabetes’ patients selectively engaging in different treatments can empower them to navigate their illness to address multiple needs [34], our study highlights a clear discontent with medical services which leads to ‘doctor shopping’ and has a knock-on effect as to how diabetes is managed.

A consequence of lack of trust in health practitioners and high costs was people would frequently miss or reduce doses of medication. Findings from other studies find that poor medication adherence is due to multiple reasons including poor health literacy, poor communication with physicians and a lack of involvement in the decision making process [35], lack of social support [36], lack of integrated care, patient beliefs and costs [30]. While it is clear that there are complex reasons for poor medication adherence, in our study decisions were not made in negotiation with a medical practitioner as relationships were more of instruction and following ‘rules’. The qualitative data found non-allopathic remedies taken for diabetes commonplace while in the survey use of non-allopathic medication was low. Survey participants may have been reluctant to admit to use. Additionally the qualitative data, similar to other qualitative research in Bangladesh [37], found that remedies and plants for diabetes was often consumed interchangeably with food – thus they may not be reported as ‘medicine’. Like other studies in South Asia our qualitative data also found that diabetes patients frequently engage with allopathic and non-allopathic treatment concurrently [31, 34, 37]. While reasons for non-allopathic medicine use are complex rooted in socio-cultural beliefs and contexts its unregulated nature means use could have safety implications.

### Limitations and strengths

The large, community-based, exclusively rural and random sample was a strength of our quantitative study. Our survey did not distinguish between type 1 and type 2 diabetes, but it is reasonable to assume most instances of diabetes in our population age group and context would be type 2. Awareness of one’s diabetic status was an inclusion criteria for our measures of care-seeking in the survey and to be interviewed. Individuals with diabetes who report a prior medical diagnosis are in the minority and it is likely that they are systematically different from the majority of people living with diabetes in rural Bangladesh. Barriers to care-seeking and glycaemic control might be different for other people with diabetes should they become aware of their status. Similarly, self-reported measures of use of care and treatments and experiences of complications may also be subject to information bias. The qualitative researcher who undertook data collection was from BADAS, which could mean research participants were reluctant to be critical of services. To mitigate this, she made clear anonymity of participants, her role as a researcher and that anything said would not have negative consequences. The assessment of quality of care was from a patient perspective meaning concerns could not always be verified and we were not able to assess efficiency of care. However, understanding patients’ perspectives and concerns, triangulated with health worker data, was a strength of the study. Additionally, having complimentary quantitative and qualitative data was a considerable strength of the study, as the qualitative data was able to explain the quantitative findings (such as the differences between the wealth quintiles) and explore the nuances missed in the survey (such as medication adherence and gender differences).

## Conclusion

While people with known diabetes seek and receive care in rural Bangladesh, the care is often suboptimal, rarely personalised and inequitable which can have serious implications on health outcomes. Diabetes is frequently diagnosed late, and many people with diabetes have complications, necessitating specialised care which is difficult to access. People with diabetes have to navigate specialists services that are hard to access, seeking care from a more trusted more local non-specialist, and managing their diabetes at home with little input from health care providers.

It is important that health care respond to local needs and understand the drivers of health-seeking [31]. At a primary care-level health professionals need to be better equipped to diagnose and manage diabetes with clear guidelines and training. The training of non-specialists in the identification, management and care of diabetes [38] could be a way of improving diabetes care locally. Strengthening PHC in communities is in-line with WHO PEN interventions [11] if implemented effectively would mean non-specialists are better equipped to care for people with diabetes. There is a clear need for more personalised care and a shift in power with patients being able to openly discuss the management of their diabetes and medication practices without fear of judgement, however this would need a much longer term change in culture and the practice of medicine. Furthermore inequitable access to care and the disproportionate negative affect on the poorest require much wider socioeconomic and policy level changes. However making effective care more accessible through local health services will help improve access to care. The government of Bangladesh’s multi-sectoral plan for NCDs and the commitment to scale up WHO PEN [12] is encouraging, and if implemented at scale should play a vital role in strengthening PHC and services for people with diabetes.

## Data Availability

The data sets used and analysed for this study may be available from the D-Magic study principle investigator (EF) upon reasonable request.
